# The Role of Viral Proteins in the Regulation of Exosomes Biogenesis

**DOI:** 10.3389/fcimb.2021.671625

**Published:** 2021-05-13

**Authors:** Xiaonan Jia, Yiqian Yin, Yiwen Chen, Lingxiang Mao

**Affiliations:** ^1^ Department of Laboratory Medicine, The Affiliated People’s Hospital, Jiangsu University, Zhenjiang, China; ^2^ Department of Immunology, Jiangsu Key Laboratory of Laboratory Medicine, School of Medicine, Jiangsu University, Zhenjiang, China

**Keywords:** virus, regulate, exosomes, biogenesis, extracellular vesicles

## Abstract

Exosomes are membrane-bound vesicles of endocytic origin, secreted into the extracellular milieu, in which various biological components such as proteins, nucleic acids, and lipids reside. A variety of external stimuli can regulate the formation and secretion of exosomes, including viruses. Viruses have evolved clever strategies to establish effective infections by employing exosomes to cloak their viral genomes and gain entry into uninfected cells. While most recent exosomal studies have focused on clarifying the effect of these bioactive vesicles on viral infection, the mechanisms by which the virus regulates exosomes are still unclear and deserve further attention. This article is devoted to studying how viral components regulate exosomes biogenesis, composition, and secretion.

## Introduction

Exosomes are a subset of extracellular vesicles (EVs) with endocytic origins (Kalluri, 2016; van Niel et al., 2018), formed during the maturation of endosomes and upon the invagination and budding of the limiting membrane of late endosomes to form intraluminal vesicles (ILVs), also known as multivesicular bodies (MVBs). Exosomes are differentiated by their size (~40–160 nm) and the presence of specific surface markers such as TSG101, Alix, Flotillin-1, CD9, and CD63, all of which distinguish exosomes from other extracellular vesicles (Théry et al., 2018). They are secreted by almost all living cells, under certain physical and pathological conditions, into various biological fluids, such as urine (Zhou et al., 2008), blood (Chiara et al., 2013), breastmilk (Brinton et al., 2015; Qin et al., 2016), and saliva (Joseph et al., 2012; Brinton et al., 2015). Exosomes carry a variety of active components, such as lipids, proteins, mRNAs, and microRNAs, derived from their parental cells. Surface receptors on exosomes allow them to be targeted to and captured by recipient cells. In addition, exosomes can be selectively taken up by neighboring and distant cells, acting as important factors involved in the regulation of intercellular signaling and biological function (Valadi et al., 2007; Raposo and Stoorvogel, 2013; Yim et al., 2016).

Exosomes are a product of the endocytic pathway, whereby early endosomes are formed by the inward budding of the plasma membrane, mature into early endosomes, and develop into late endosomes, which are characterized by the formation of ILVs within the late endosome, also known as MVBs. MVBs fuse with the plasma membrane and release ILVs into the extracellular milieu, as exosomes. Alternatively, MVBs move towards lysosomes resulting in the degradation of the MVBs and their encapsulated components. The initial function of exosomes is to expel intracellular metabolic waste into the extracellular milieu, which is analogous to a cellular waste removal system (Johnstone et al., 1987; Johnstone et al., 1989). With further research, it has been proved that exosomes could modulate cell-cell communication by transferring bioactive substances. The endosomal sorting complex required for transport (ESCRT) is a conserved complex of proteins that regulates the deformation and scission of lipid membranes, induces the formation of ILVs and exosome secretion, and has been shown to facilitate vesicle packaging by ubiquitination (Slagsvold et al., 2005; Mayers et al., 2011; Agromayor et al., 2012). There is also evidence of an ESCRT-independent pathway-directed exosomes biogenesis, mediated by ceramide, phospholipase D2 (PLD2), ADP ribosylation factor-6 (ARF6), and the tetraspanins CD63 and CD81 (Kosaka et al., 2010; Ghossoub et al., 2014).

Besides exosomes, cells can also secrete other types of vesicles including microvesicles, ectosomes, and apoptotic bodies. The accurate isolation and purification of exosomes, to distinguish them from potential vesicular contaminants, is an essential aspect of exosomal research. Methods used in the isolation of exosomes include ultracentrifugation, ultrafiltration, chromatography, polymer-based precipitation, affinity capture, and so on. Ultracentrifugation, which comprises both differential ultracentrifugation and density gradient centrifugation, is a well-established, time-consuming method requiring costly equipment. Ultracentrifugation can reach speeds up to 100,000 g and is most suitable for large-scale rather than small-scale clinical sample preparations. Ultrafiltration involves the extraction of exosomes on the basis of their size and molecular weight (Mw), and is capable of isolating all molecules with an Mw < 100 kDa cut-off. This method can be used to concentrate a large sample volume for subsequent purification. Size-exclusion chromatography (SEC), which separates particles according to size, albeit by exploiting commercially available membranes, can yield pure vesicles. However, exosomes fractions isolated by SEC are still contaminated with protein aggregates. The polyethylene glycol-based precipitation method causes exosomes to precipitate out by changing their solubility and dispersibility, thus extracting the exosomes from the sample simply and quickly by nonspecific means. The affinity capture technique relies on magnetic beads coated with specific antibodies and enables the separation of exosomes expressing particular antigens in a highly specific manner. While this method is rapid, easy to perform, and can yield specific subsets of exosomes, it remains expensive (Gurunathan et al., 2019; Zhang et al., 2020). The fact that exosomes are similar in size, shape, and density to many viruses makes separating exosomes from viruses a veritable challenge. Typically, exosomes are purified from the virus infected culture medium using a combination of ultracentrifugation and density gradient centrifugation (Cantin et al., 2008; Mori et al., 2008; Liu et al., 2014; Zhu et al., 2015; Jiang et al., 2020) or magnetic bead-mediated immunoaffinity capture (Fu et al., 2017). Generally, a combination of appropriate exosome separation methods is recommended, whereby one method is used to concentrate, and another method is used to purify the exosomes. As no standard method for the isolation of exosomes exists to date, we should recognize that the exosomes in early researches may be contaminated by trace amounts of other type vesicles, and that the different biological markers in exosomes may relate to different subpopulations of exosomes with specific compositions.

Recent studies have recognized that exosomes secreted by infected cells play a dual role by both enhancing and inhibiting viral infection. For example, exosomes carrying viral infectious nucleic acid facilitate viral transmission between cells. Exosomes from the HCV-infected human hepatoma cell line Huh7.5.1, containing the full-length viral RNA genome, were transferred to uninfected Huh7.5.1 cells, establishing productive infection in a virion-independent manner (Ramakrishnaiah et al., 2013). As a result, exosomes can be viewed as an alternate form of the virus and a source of infectious particles. In addition, virions cloaked in membrane vesicles can evade the immune system efficiently. This form of ‘membrane wrapping’ protects these viruses from neutralizing antibodies while facilitating the spread of viruses such as Coxsackie B virus (Sin et al., 2017) and enterovirus 71 (Mao et al., 2016). On the other hand, exosomes can also limit the spread of virus through a variety of mechanisms. For instance, exosomes from HCV-infected cells were able to transfer viral RNA to plasmacytoid dendritic cells (pDCs) and trigger the production of antiviral type 1 interferons (IFNs), initiating the host innate immune response against the virus (Dreux et al., 2012). In addition to the viral components, exosomes contain various proteins derived from their parental cell, including host proteins with antiviral activity and the ability to resist infection (Li et al., 2013; Zhu et al., 2015). For instance, the apolipoprotein B mRNA editing enzyme catalytic subunit 3G (APOBEC3G), a cytidine deaminase and a critical component of the anti-retroviral defense system, was transferred into adjacent cells to inhibit HIV-1 replication *via* exosomes (Khatua et al., 2009). Furthermore, exosomes secreted into the airways of mice have been shown to incorporate host proteins with anti-influenza activity as well as viral proteins which are able to trigger host immune responses (Bedford et al., 2020).

There are a number of studies on the role of exosomes in the context of viral infection, but few studies have focused on the mechanisms by which the virus regulates the production of exosomes and determines their contents. Cargoes sorted into the exosomes are highly dependent on the physiological and pathological state of the cell. When viruses infect cells, they hijack the endosomal pathway and incorporate the viral components into the exosomes. Mechanistically, the viral component leads to changes in the formation and secretion of exosomes. This review provides a comprehensive summary of the viral components involved in regulating exosomes biogenesis, composition, and secretion. To the best of our knowledge, this review for the first time discusses the mechanisms employed by viral components to regulate the secretion and sorting of exosomes.

## RNA Viruses

### Human Immunodeficiency Virus

The human immunodeficiency virus (HIV), responsible for acquired immunodeficiency syndrome (AIDS), is a lentivirus belonging to the *Retroviridae* family. Nef is a nonenzymatic accessory protein with multiple functions, used by HIV to establish the infection of host cells.

HIV-1 infection has been shown to increase the production of exosomes from infected macrophages (Kadiu et al., 2012), and Nef expression appears to significantly increase the number of late endosomes/MVBs (which account for exosomes biogenesis) in various cell lines, including human T lymphocytes (Sanfridson et al., 1997), HeLa, CIITA, and Mel JuSo cells (Stumptner-Cuvelette et al., 2003). Considering that the increase in MVB production may lead to a rise in the release of ILVs, these results suggest that Nef may be involved in exosomes biogenesis. Indeed, several studies have found that the Nef protein was able to enhance exosome production in astrocytes (Pužar Dominkuš et al., 2017), CD4^+^ T cells (Lenassi et al., 2010), and U937 cells (Aqil et al., 2014). The specific amino acids sequence and domain of Nef, which is responsible for the Nef-induced vesicle release have been discovered (Ali et al., 2010). However, de Carvalho et al. reported that Nef made no significant difference to exosome secretion by CD4^+^ T cells (de Carvalho et al., 2014). Therefore, the role of Nef in regulating exosome secretion requires further elucidation.

HIV-1 infection also alters the exosomal proteome (Li et al., 2012), and it has been observed that Nef plays a certain role in changing the composition of exosomes. First and foremost, Nef is incorporated into the exosomes from HIV-infected cells (Muratori et al., 2009) and Nef-transformed T cells (Campbell et al., 2008; Lenassi et al., 2010). Nef stimulates itself to be released from cells *via* the exosomal pathway (Lenassi et al., 2010). Functionally, exosomal Nef can induce activation-induced cell death of bystander CD4^+^ T lymphocytes, which is a key factor involved in progression to AIDS (Lenassi et al., 2010), and trigger inflammation through impairing cholesterol metabolism and enhancing the abundance of lipid rafts in bystander cells (Mukhamedova et al., 2019). Besides, Nef promotes HIV-1 infection through the sequestration of CD4 and MHC class 1 molecules into MVBs to degradation, because the presence of CD4 molecules on the exosomal surface can inhibit viral transmission through adhesion to the viral envelope (de Carvalho et al., 2014). Proteomic analysis has indicated that exosomes secreted by HIV-infected T lymphocytes upregulated ADP-ribosyl cyclase 1 (CD38) and downregulated Annexin A5 (ANXA5) and L-lactate dehydrogenase B chain (LDHB). CD38, ANXA5, and LDHB have been shown to interact with the HIV viral proteins Tat and p24, and are thought to be relevant to apoptotic and proliferative cellular processes (Li et al., 2012). Despite these reports, the exosomal sorting mechanism and the specific roles of these proteins during viral infection are still unknown.

Nef cannot only affect the sorting of protein molecules into exosomes, but is also implicated in the sorting of microRNAs (miRNAs) into exosomes. Analysis of the miRNome suggests that the expression of Nef protein in human monocytic U937 cells contributes to the redistribution of miRNA between cells and exosomes. Compared to the exosomes secreted by the control group, miRNAs incorporated into exosomes secreted by Nef-expressing cells were upregulated. Besides, the expression of Nef caused 2 miRNAs to remain within the cell, while 47 miRNAs were sorted into Nef exosomes. Interestingly, miRwalk software analysis revealed that the miRNAs that were upregulated in the exosomes (such as miR-146a, miR-146b-3p, miR-125b, and miR-181a), targeted innate immune responses, rendering parent U937 cells more permissive to viral replication and persistence. However, the mechanism by which Nef selectively regulates miRNA sorting into exosomes is still unclear, and how the Nef-mediated exosomal miRNAs affect the recipient cells are still important questions to be explored in future research (Aqil et al., 2014).

### Ebola Virus

The Ebola virus (EBOV) is an enveloped, single-stranded RNA virus from the family *Filoviridae*, known to cause severe hemorrhagic fever with up to 80–90% mortality rates observed in both humans and non-human primates. The EBOV matrix protein VP40, composed of 326 amino acids (35 kDa), is the most abundant matrix protein contained in mature EBOV virions. VP40 is not only packaged into exosomes that negatively impact on recipient immunocytes (Pleet et al., 2016; Pleet et al., 2017), but also affects exosomes biogenesis and release. Transfection of EBOV VP40 was shown to increase intracellular levels of the exosomal marker CD63 and the production of exosomes, while the treatment of VP40-resistant cells with the FDA-approved drug oxytetracycline diminished exosomes release. In-depth research showed that VP40 upregulated intracellular levels of specific ESCRT machinery components (the ESCRT-I protein TSG101, and the ESCRT-II proteins EAP20 and EAP45) and CD63, which was accompanied by the upregulation of glycosylated CD63 and Alix protein levels within the exosomes. Considering that exosomes biogenesis depends to a large extent on the ESCRT pathway, the ESCRT machinery components upregulated by VP40 may be responsible for the increase in exosome production. Besides this, the presence of VP40 was shown to cause a reduction in some miRNA machinery components including Dicer, Drosha, Ago1, and DGCR8 in donor cells. Consequently, VP40-containing exosomes lowered the protein levels of Dicer, Drosha, and Ago 1 in the recipient lymphocytes, resulting in cellular dysfunction and viral persistence (Pleet et al., 2016). Subsequently, the in-depth study of the relationship between VP40 and exosomes biogenesis by this team suggests that VP40 is able to alter exosomes biogenesis through the induction of cell cycle dysregulation. They found that EVs isolated from VP40-expressing cells were enlarged but less numerous. Chromatin Immunoprecipitation and PCR analyses of Cyclin D1 Promoter DNA showed that VP40 upregulated cyclin D1 by binding to the cyclin D1 promoter, resulting in accelerated cell cycling and in turn impacting on exosomes biogenesis. Employing specific inhibitors of cyclin D1 kinase activity led to a significant increase in the number of exosomes produced by VP40-expressing cells. It is suggested that VP40 affects exosomes biogenesis and secretion in a cell cycle-dependent manner. VP40 markedly altered the expression patterns of ESCRT proteins (increased levels of TSG101, CHMP6, and EAP20), exosomal markers in EVs (increased level of Alix and CD63), and the amount and morphology of EVs at G0 phase in particular. However, these changes were not as evident in VP40-expressing 293T cells during the G1/S or G2/M phases. In summary, EBOV VP40 modulates EV biogenesis including the quantity, morphology, and content of EVs *via* cell cycle acceleration in donor cells (Pleet et al., 2018). As for the effect of VP40 on exosomes biogenesis, the somewhat contrasting nature of the above two results, indicates that this area of research would benefit from further exploration.

### Hepatitis A Virus

Recently, it has been reported that the supernatant of cells infected with the hepatitis A virus (HAV) contained two populations of virus particles: ‘naked’ virus particles and membrane-wrapped HAV particles (~50 to 110 nm). These membranous vesicles containing viral RNA and protein resemble exosomes and are called as quasi-enveloped HAV (eHAV). It was reported that the eHAV form is present in the culture medium of HAV-infected cell lines, including Huh-7.5 human hepatoma cells (Feng et al., 2013) and human colonic adenocarcinoma-derived (Caco-2) cells (Hirai-Yuki et al., 2016). While eHAV particles have a density of ~1.05–1.10 g/cm^3^ and range from ~50 to 110 nm in diameter, which is reminiscent of exosomal dimensions, nonenveloped HAV particles have a density of ~1.27 g/cm^3^ and are ~27 nm in diameter (Feng et al., 2013). In addition, only eHAV (and not nonenveloped HAV) can be detected in serum samples from patients with early stage acute hepatitis A infection. The membrane envelope protects the viral protein core from the human immune response, rendering eHAV resistant to neutralizing antibodies, circulating in the host, and then facilitating its dissemination in the liver (Feng et al., 2013). Proteomic analysis of this unconventional secretion of eHAV revealed that eHAV particles possess HAV capsid proteins but lack nonstructural proteins. These infectious eHAV vesicles are also enriched in endolysomal components including the exosome-specific marker CD9 and the dipeptidyl peptidase 4 (DPP4), but lack markers of autophagy. In addition, RNAi-mediated knockdown of ESCRT-III associated proteins including Alix, VPS4B, and CHMP2A could effectively ablate eHAV release. These results indicate that HAV particles were selectively packaged as cargoes into membranes *via* an exosome-like sorting mechanism, forming eHAVs (McKnight et al., 2017).

The structural protein pX is an unusual carboxy-terminal 71 amino acid (8.3 kDa) extension on VP1 that among picornaviruses is unique to HAV. The HAV P1-2A capsid protein precursor is cleaved by 3Cpro to generate VP0, VP3, and VP1-pX. VP1-pX is then cleaved by an as yet unidentified cellular protease to remove the pX protein and release the mature capsid protein VP1. Interestingly, pX was found to be present only in eHAV particles but not in nonenveloped virions (Feng et al., 2013). Subsequently, researchers found that pX was involved in cargo sorting into membranous vesicles and direct foreign protein into MVBs. When the human hepatocellular carcinoma cell line Huh7 was transfected with the plasmid encoding the recombinant eGFP-pX protein, eGFP-pX was located within the exosomes, while cells expressing only eGFP could not secrete exosomes containing eGFP. This implies that pX, when fused to eGFP was directly responsible for guiding eGFP secretion into the exosomes. In addition, 3D live cell imaging clearly showed that the cellular eGFP-pX puncta signal was colocalized with Alix and CD63, and that the diameter of colocalization puncta corresponded to MVBs (~400 nm), suggesting that eGFP-pX was contained within MVBs from which the exosomes then originated. Furthermore, researchers found that the C-terminal domain (amino acids 31–71) of pX could interact with Alix to promote the loading of eGFP into exosome-like vesicles. Analogously, when an infectious HAV clone in which the 36 C-terminal amino acids (32–67) of pX had been deleted, was used to infect Huh7 cells, it was found that eHAV production decreased dramatically, indicating that this C-terminal section of pX could regulate eHAV secretion by affecting the interaction between HAV and Alix (Jiang et al., 2020). Collectively these data point towards the strong possibility that pX is a critical factor in promoting the selective sorting of HAV particles into exosomes.

## DNA Viruses

### Herpesvirus

Herpesviruses are large, enveloped, double-stranded DNA (dsDNA) viruses that are organized into three families (alpha, beta, and gamma), according to their biological properties. Of these, eight herpesviruses infect humans including three alphaherpesviruses (herpes simplex virus 1, herpes simplex virus 2, and Varicella-Zoster virus), three betaherpesviruses (human cytomegalovirus, human herpesvirus-6, and human herpesvirus-7), and two gammaherpesviruses (Epstein-Barr virus and Kaposi’s sarcoma–associated herpesvirus). Herpesviruses consist of a toroid-shaped DNA genome wrapped in an icosahedral capsid with 162 capsomers, surrounded by a proteinaceous tegument and an outer lipid bilayer envelope containing different glycoprotein spikes. The characteristic of this group of viruses is that they can establish life-long latency after primary infection. The viral genome is maintained and distributed to progeny cells that are not infected with the virus. And the expression of viral genes is restricted.

### Human Herpesvirus 1

Human herpesvirus 1(HHV-1), also called Herpes simplex virus (HSV-1), is a member of the subfamily *Alphaherpesvirinae*. The virus infects mucoepithelial cells in the early stage and establishes a latent viral pool in the neurons of sensory ganglia, which mostly occurs in infancy and is usually asymptomatic. Researchers found that HSV-1-infected cells were able to secrete CD63-positive EVs/exosomes containing viral and host factors that negatively impacted on HSV-1 infection (Kalamvoki et al., 2014; Deschamps and Kalamvoki, 2018). HSV-1 infection promotes the release of CD63-positive EVs accompanied by a decrease in intracellular CD63 expression levels. These CD63-positive EVs can restrict viral dissemination to help HSV-1 persistence within the host (Dogrammatzis et al., 2019). Although the concrete mechanism underlying the production of these CD63-positive EVs is not yet clear, the HSV-1 γ134.5 neurovirulence gene could affect the release of the exosomes. Heikkilä et al. showed that exosome marker proteins were abundant in the wildtype HSV-1 fractions separated by ultracentrifugation and density gradient centrifugation, whereas they were undetectable in the HSV-1 fractions harboring a γ134.5 deletion, indicating that deletion of the viral γ_1_34.5 gene markedly attenuated HSV-induced exosomes secretion (Heikkilä et al., 2016). The deletion of the HSV-1-specific γ_1_34.5 gene thus renders the virus non-neurovirulent (Mattila et al., 2015) and has been employed in cancer therapy (Hu et al., 2006). It is recommended that the purity of γ134.5 depleted HSV-1 stocks is however verified to avoid the presence of contaminating exosomes before its use in gene therapy.

Glycoprotein B (gB) is one of the most highly conserved envelope glycoproteins among members of the *Herpesviridae* family and plays an important role in *alphaherpesvirus* infection. Immunofluorescence analysis has shown that gB localizes with MVB membranes in HSV-1-infected and gB-expressing 293T cells (Calistri et al., 2007). Glycoprotein B can be detected in the exosomes from gB-expressing cells, meaning that it is transported to MVBs and subsequently released by exosomes (Temme et al., 2010). The MVB pathway is essential not only for viral assembly and virion budding but also for the biogenesis of exosomes, suggesting that gB is likely to be directly involved in the formation and secretion of exosomes.

Recent studies have reported that gB actually participates in exosome cargo sorting. In antigen-presenting cells (APCs), the human leukocyte antigen DR (HLA-DR) molecule, a molecular component of the MHC class II pathway, binds to antigen in MHCII-loading compartments. Then, these MHCII heterodimers are loaded onto the cell surface for antigen presentation through the back-fusion between MVB and the cell membrane, resulting in immunity against HSV-1 infection. Previous studies have shown that gB was co-isolated with HLA-DR in HSV-1-infected cells, indicating that gB may play a role in antigen presentation (Neumann et al., 2003). However, Temme et al. found that gB-DR complexes could not be detected on the cell surface in gB-transfected cells. In cells expressing gB, gB is able to bind to DR and inhibit the binding of antigenic peptide to the DR portion of the complex. Glycoprotein B, DR, and CD63 colocalize in the endosome-derived large intracellular vesicles, which may affect the biogenesis and trafficking of MVBs. Moreover, the gB-DR complexes can be detected in exosomes and the significant ubiquitination of gB but not the DR subunits was detected. These results suggest that the ubiquitination of gB is likely to participate in sequestering DR into the exosomes instead of targeting to the cell surface. Thus, the viral protein gB binds to DR and the ubiquitinated gB transfers DR into the exosomes, hindering the presentation of viral antigens *via* the classical MHC class II processing pathway, which may represent a novel immune escape mechanism employed by viruses (Temme et al., 2010).

### Human Herpesvirus 4

Human herpesvirus 4, also known as Epstein–Barr virus (EBV), was the first human tumor-associated virus to be identified, due to the close link between EBV infection and nasopharyngeal carcinoma (NPC). EBV-infected cells have been shown to secrete exosomes. EBV infection (type III latently infected cells in particular) was shown to upregulate the biogenesis of exosomes and promote specific miRNA sorting into exosomes (Nanbo et al., 2018). Latent membrane protein 1 (LMP1), an EBV-encoded oncoprotein, is a transmembrane glycoprotein composed of 386 amino acids (41 kDa) that has been shown to be sorted into exosomes in a CD63-dependent manner (Hurwitz et al., 2017). Besides, component of the ubiquitin system plays a role in the transport of LMP1 into the exosomes (Kobayashi et al., 2018). Functionally, exosomal LMP1 can trigger the proliferation and invasion of tumor cells *in vitro* and enhance tumor progression *in vivo* (Liao et al., 2020).

LMP1 was proven to be involved in the regulation of exosomal secretion. Increasing evidence indicates that the expression of LMP1 can promote the secretion of extracellular vesicles, especially exosomes (Verweij et al., 2011; Hurwitz et al., 2017). To this end, researchers have studied the regulatory mechanism employed by LMP1 in exosomes biogenesis. They found that LMP1 can activate the transcription nuclear factor (NF)-kappa B signaling pathway, and the activated NF-kappa B caused increased expression levels of two downstream target genes, syndecan2 (SDC2) and synaptotagmin-like-4 (SYTL4) directly in NPC cells. Inhibiting the expression of SDC2 in LMP1 was shown to reduce the formation of MVBs and thus impair the secretion of exosomes. According to previous research, the interaction of SDC2 and syntenin is crucial for the syndecan-syntenin-Alix exosomes biogenesis pathway, which has been proven to promote exosomes biogenesis and exosomal cargo sorting (Baietti et al., 2012; Roucourt et al., 2015). Liao et al. found that the interaction between SDC2 and syntenin was enhanced by the expression of LMP1. Meanwhile, inhibition of STYL4 in LMP1 expression may be due to the inhibition of the fusion of MVBs to the plasma membrane, resulting in a large amount of MVB accumulation in the cell and diminished exosomal secretion. Combined with the above, SDC2 and STYL4 are the key components that lead to the increase of exosome secretion mediated by LMP1. Thus LMP1 can increase the amount of EV which would enhance the proliferation and invasion of recipient NPC cells to aggravate NPC progression (Liao et al., 2020).

### Human Herpesvirus 6

Human herpesvirus 6 (HHV-6) is a betaherpesvirus and is divided into two species: HHV-6A and -6B. HHV-6 infection can cause encephalitis in immunocompromised hosts and as well as other disorders of the central nervous system, such as Alzheimer’s disease (Greninger et al., 2018). Electron microscopy analyses of HHV-6-infected cells have revealed the presence of larger numbers of newly formed MVBs (including small vesicles in the cytoplasm) compared to uninfected cells. The HHV-6 envelope gB and CD63 proteins colocalize in MVBs, implying that gB might be involved in MVB formation and affect exosomes biogenesis in the context of HHV-6 infection (Mori et al., 2008).

### Hepatitis B Virus

Hepatitis B virus (HBV) is a hepatotropic DNA virus of the *Hepadnaviridae* family of enveloped pararetroviruses, which causes liver diseases such as chronic hepatitis and hepatocellular carcinoma (HCC). HBV X (HBx), a small non-structural X protein composed of 154 amino acid residues (16 kDa), is encoded by the smallest open reading frame (X) of the HBV genome. Numerous studies show that HBx is responsible for multiple roles in the development of HBV-related liver cancer, and is involved in regulating multiple hepatocyte signaling processes such as apoptosis (Miao et al., 2006; Clippinger et al., 2009; Elizalde et al., 2017), the dysregulation host miRNA expression (Zhang et al., 2013), and the inhibition of nucleotide excision repair of damaged cellular DNA (Martin-Lluesma et al., 2008). Collectively, the disruption of normal cellular processes orchestrated by HBx provides favorable conditions for virus replication.

HBx is confirmed to regulate exosomes biogenesis. Kapoor et al. found that there was a significant increase in the number of exosomes secreted by HBx-expressing Huh-7 cells compared to control cells (Kapoor et al., 2017). To investigate this mechanism, they showed that HBx markedly (> 2 fold) increased the activity of neutral sphingomyelinase 2 (nSMase2). In addition, co-immunoprecipitation results showed that various exosomal markers including CD9, CD81, and nSMnase2 proteins can interact with HBx. And immunofluorescence provided evidence for the co-localization of HBx with CD81 and CD63, respectively. Since nSMase2 is a key enzyme involved in exosome production and secretion (Dreyer and Baur, 2016), and tetraspanin CD63, CD9, and CD81 are major components of exosomes biogenesis (Chairoungdua et al., 2010; van Niel et al., 2011), HBx is likely to increase exosomes secretion by promoting nSMase2 activity and interacting with CD9, CD81, and nSMase2. Indeed, exosomes containing biologically active HBx mRNA and protein have been shown to induce the proliferation of hepatic stellate cells (HSCs) (Kapoor et al., 2017). However, the mechanism by which HBx is sorted into exosomes remains unknown, and in-depth studies are required to further elucidate the underlying details.

Recent studies suggest that the expression of HBx in Huh-7 cells decreases the intracellular levels of the classical host antiviral factor APOBEC3G (A3G). The A3G protein is widely known as a restrictor factor of HBV replication because A3G can block HBV infection by inhibiting the accumulation of cellular viral longer minus-strand DNA (Nguyen et al., 2007). Researchers found that HBx-expressing cells displayed significantly lower levels of cellular A3G independently of proteasome and lysosomal degradation processes. Furthermore, increased intracellular A3G levels were observed following the inhibition of the exosomal pathway, meaning that intracellular A3G can be exported *via* the production and secretion of exosomes. A further study showing that HBx-expressing cells are associated with heightened A3G protein levels within the exosomes implies that HBx manipulates A3G protein levels through the transportion of A3G into exosomes and its secretion into the extracellular environment. Therefore, HBx-induced exosomal A3G export is likely responsible for the decreased intracellular A3G protein levels observed during HBV infection (Chen et al., 2017). The mechanism of HBx-mediated A3G sorting into exosomes is worth pursuing.

### Prions

A prion is a self-replicating and infectious factor composed of prion protein (PRNP) in the absence of nucleic acid. PRNP is a glycoprotein abundantly expressed in neuronal cells but can also occur in non-neuronal cells. Prion diseases arise following conformational changes that convert the PRNP PrPC to its pathological isoform PrPSc. The accumulation of PrPSc in nervous tissue causes several severe fatal neurodegenerative diseases in humans and animals. Further, prions can act as infectious agents that cause transmissible spongiform encephalopathies.

While we know that prions can be packaged into endosome-derived exosomes to infect neighboring cells (Vella et al., 2007; Coleman et al., 2012), the molecular and cellular mechanisms are not fully understood. Dias and colleagues found that, compared with wildtype mice, astrocytes and fibroblasts collected from PRNP-null mice experienced an activated autophagy event (aggregate of autophagosomes and phagophore), a lack of MVBs in the cytoplasm, and impaired exosome secretion. Considering that a close relationship exists between autophagy and exosomes biogenesis (Fader et al., 2008), they explored the mechanism by which PRNP could regulate exosomes biogenesis through the autophagy pathway. The results showed that the inhibition of autophagy activation by knocking down the BECN1 gene increased exosomal secretion significantly in PRNP-deficient astrocytes, whereas recovery of PRNP expression at the cell membrane could restore exosome secretion. This indicates that the high correlation between abnormal autophagy events and subsequent exosome secretion is regulated by PRNP. Furthermore, the octapeptide repeat domain of PRNP was found to be responsible for attenuating autophagic flux and increasing exosome secretion. Next, the research group found that PRNP could promote the translocation of CAV1 into the cytoplasm, causing the breakdown of the ATG5-ATG12 interaction in the cytoplasm, inhibiting autophagy (Chen et al., 2014) and ultimately promoting exosome secretion. As such, it was concluded that PRNP facilitates the trafficking of MVBs containing ILVs towards the plasma membrane to release exosomes rather than direct them towards lysosomes for degradation (Dias et al., 2016). Recently, another group confirmed that PRNP could be trapped in the exosomes of prion-infected neuronal cells, and that autophagy was shown to modulate the secretion of these PRNP^+^ exosomes in two different neuronal cells (CNS-derived ScCAD5 cells and peripheral nervous system neuronal cells ScN2a) (Abdulrahman et al., 2018). However, the difference in the extent of exosomal secretion observed in the ScCAD5 and ScN2a cells does not appear to be related to PRNP level, and therefore would merit from further study.

## Conclusion

The consensus among scientists is that viral infections can regulate the biogenesis of exosomes, which plays a role in viral transmission and pathogenesis. For example, infection with the respiratory syncytial virus (RSV) induces cells to secrete more exosomes and change exosomal components. These exosomes, containing the RSV nucleocapsid protein N, attachment protein G, fusion protein F, and viral RNA, were shown to induce cytokine and chemokine secretion by human monocytes and airway epithelial cells instead of transmitting RSV infection (Chahar et al., 2018). Protein composition changes have also been observed in airway exosomes during influenza virus infection. Increasing levels of host proteins targeting the influenza virus and four influenza virus proteins (HA, NS1, NP, and M1), were detected in the airway exosomes isolated from influenza virus-infected mice. However, how these host proteins with antiviral activity are sorted into exosomes and whether they are associated with the viral proteins inside the exosomes, needs to be further explained (Bedford et al., 2020). Zika virus (ZIKV) infection causes increased exosomal production, with the incorporation of ZIKV RNA and ZIKV E protein into the exosomes (Zhou et al., 2019). Besides these, the exosomes secreted by HCV-, enterovirus 71 (EV71)-, and foot-and-mouth disease virus (FMDV)-infected cells were shown to encapsulate the corresponding viral nucleic acid, and mediate viral transmission to recipient cells (Dreux et al., 2012; Mao et al., 2016; Zhang et al., 2019). Exosomes from human T cell lymphotropic virus (HTLV)-infected cells were also found to contain the HTLV Tax protein and HTLV-1 mRNA transcripts (encoding *env*, *tax*, and *hbz*), which protect the recipient cells from apoptosis (Jaworski et al., 2014). However, the specific mechanisms implicated in the sorting of these viral exosomal components are not clear and require further research.

Viruses hijack the endosomal pathway or the endosomal machinery for their own benefit: not only for the production of new virions, but also for immune evasion or to facilitate viral spread. Many studies have suggested that the virus can hijack the exosomal pathway to facilitate virus budding, aggregation, or release, and that the hijacked exosomes assist the virus in escaping immune recognition and promote its dissemination. Importantly, there are many research reports that document that virus infected cells release more exosomes and are able to alter the exosomal content. Furthermore, individual virus components can affect the cargo loaded into exosomes independently of the intact viral particles themselves, which is sufficient to change the physiological environment of recipient cells. Here, we emphasize the role of and the cellular mechanisms employed by various viral components in the biogenesis and secretion of exosomes (summarizes in **Table 1**). However, the impact of viral infections on exosomes has not been fully explained. Clarifying how the viral component affects exosomes biogenesis and what impact these exosomes have on the recipient cells will contribute to the development of novel therapeutic targets for viral infections.

**Table 1:** The viral proteins known to regulate exosomes biogenesis.

Virus type	Virus name	Viral components	Effector	Function and mechanism	Reference
RNA VIRUS	HIV-1 (+ssRNA)	Accessory protein Nef	—	Nef expression reduces the sorting of CD4 and MHC class 1 molecules into exosomes	(de Carvalho et al., 2014)
				Nef expression in monocytes alters miRNA distribution within exosomes and cells	(Aqil et al., 2014)
	EBOV (-ssRNA)	Matrix protein VP40	—	VP40 may increase the production of exosomes by upregulating some ESCRT machinery components.	(Pleet et al., 2016)
			cyclin D1	VP40 reduces the number of while increasing the size of secreted EVs, especially at G0 phase, though its modulation of the cell cycle *via* upregulation cycling D1 expression.	(Pleet et al., 2018)
	HAV (+ssRNA)	Structural protein pX	Alix	PX transports foreign protein and virus particles into MVBs prior to their release *via* exosomes by interacting with Alix directly.	(Feng et al., 2013; Jiang et al., 2020)
DNA VIRUS	HHV-1 (dsDNA)	gB	DR molecules	Glycoprotein B binds to DR, and ubiquitinated gB transfers DR-gB complexes into the exosomes.	(Temme et al., 2010)
		Accessory protein γ_1_34.5	—	The deletion of viral γ_1_34.5 gene markedly attenuates HSV-induced exosome secretion.	(Heikkilä et al., 2016)
	HHV-4 (dsDNA)	LMP1	SDC2 and SYTL4	LMP1 increases exosome secretion by upregulating SDC2 and SYTL4 in cells and the interaction between SDC2 and syntenin.	(Liao et al., 2020)
	HBV (dsDNA)	HBx	nSMASE2 and CD9, CD81, and CD63	HBx increases exosome secretion by upregulating the activity and mRNA level of nSMase2 and interacting with the exosomal biomarkers CD9, CD81, and nSMase2.	(Kapoor et al., 2017);
			A3G	HBx enhances the transportation of A3G protein into the exosomes.	(Chen et al., 2017)
PRIONS	protein-based infectious agent	PRNP	Caveolin-1	PRNP increases exosome secretion by inhibiting the caveolin-1-activated autophage pathway.	(Dias et al., 2016)

In summary, exosomes are the smallest group of nanovesicles, serving as an important method for intracellular communication. There is some overlap between the processes of exosomes biogenesis, virion assembly, and egress strategies used by host cells. For example, by treating cells with the inhibitors of MVB formation (U18666A and GW4869) or exosome secretion, the release of viruses from the host cell membrane can be significantly reduced (Ramakrishnaiah et al., 2013; Nagashima et al., 2014). In turn, there have been many studies reporting that virus infection causes changes to exosome secretion (Fu et al., 2017). However, the regulatory mechanisms involved in virion-mediated exosomes biogenesis are different and need further clarification.

## Author Contributions

XJ wrote the first draft of the manuscript. YY and YC edited and revised the manuscript. This review was designed and guided by LM. Both authors read and approved the submitted version. All authors contributed to the article and approved the submitted version.

## Funding

This work was supported by the National Natural Science Foundation of China [32070182], Zhenjiang Science and Technology Program [SH2020058], and Jiangsu Province Medical Talents Program [QNRC2016453].

## Conflict of Interest

The authors declare that the research was conducted in the absence of any commercial or financial relationships that could be construed as a potential conflict of interest.
